# Thyroid doses for the Chornobyl Tissue Bank: improved estimates based on revised methodology and individual residence and diet history

**DOI:** 10.1007/s00411-024-01099-8

**Published:** 2024-12-19

**Authors:** Sergii Masiuk, Mykola Chepurny, Valentyna Buderatska, Olha Ivanova, Zulfira Boiko, Natalia Zhadan, Halyna Chornovol, Mikhail Bolgov, Viktor Shpak, Mykola Tronko, Elizabeth K. Cahoon, Stephen J. Chanock, Tetiana Bogdanova, Lindsay M. Morton, Vladimir Drozdovitch

**Affiliations:** 1 State Institution “National Research Center for Radiation Medicine, Hematology and Oncology of the National Academy of Medical Sciences of Ukraine”, Kyiv, Ukraine; 2 Institute of Hydromechanics of the National Academy of Sciences of Ukraine, Kyiv, Ukraine; 3 State Institution “V.P. Komisarenko Institute of Endocrinology and Metabolism of the National Academy of Medical Sciences of Ukraine”, Kyiv, Ukraine; 4 Division of Cancer Epidemiology and Genetics, National Cancer Institute, NIH, DHHS, Bethesda, MD USA

**Keywords:** Chornobyl, Chernobyl, Accident, Radiation dose, Thyroid cancer, Questionnaire

## Abstract

Increased thyroid cancer incidence has been one of the principal adverse health effects of the Chornobyl (Chernobyl) nuclear power plant accident. Accurate dose estimation is critical for assessing the radiation dose-response relationship. Current dosimetry estimates for individuals from the Chornobyl Tissue Bank (CTB) are based only on the limited information on their places of residence at the time of the accident and/or at the time of surgery for thyroid cancer. The present study aimed to assess whether additional residential and dietary history data collected during personal interviews would substantially impact dose estimates. This paper presents an assessment of thyroid doses from ^131^I intake for the 197 exposed individuals from the CTB with pathologically confirmed papillary thyroid cancer. Thyroid doses, which had been calculated for these individuals in 2010, were revised in this study using the recently substantially revised ‘Thyroid Dosimetry 2020 system for Ukraine’ (TDU20). In addition, residence and diet history data were collected during personal interviews with individuals for whom dosimetry-related data were scarce. The arithmetic mean of thyroid doses estimated in this study was 510 mGy (previously 700 mGy), while the median was 81 mGy (previously 120 mGy). A rather wide range of thyroid doses from zero to 11.9 Gy (previously up to 15.0 Gy) was observed among study participants. The uncertainties in doses were characterized by the geometric standard deviation of 1,000 individual stochastic doses. As a result, the geometric standard deviation varied from 1.3 to 5.3 with an overall arithmetic mean of 2.7 and a median of 2.9. This study clearly showed that the use of individual questionnaire data in dose assessment of individuals who completed personal dosimetry interviews had a noticeable impact on the thyroid dose values: the thyroid doses changed by more than 100 mGy in 31 out of 104 (29.8% of the total) individuals, while such changes due to the use of TDU20 were observed in 18 out of 104 (17.3%) individuals. Clearly, future focused studies using samples from the CTB would benefit from personal interviews to improve dose estimates. Another lesson learned from this study is that whenever a radiation accident occurs, it is important to ask affected people by health and radiation safety authorities to keep records of their own behavior and diet, and, if possible, those of their children.

## Introduction

The accident at the Chornobyl (Chernobyl) nuclear power plant (NPP) in Ukraine on 26 April 1986 resulted in the release of 1.8 × 10^18^ Bq of Iodine-131 (^131^I), one of the most radiologically significant isotopes of radioiodine. In fact, in the general public an increased risk of thyroid cancer and other thyroid diseases associated with the exposure of the thyroid gland to ^131^I in childhood and adolescence was the main health effect of the Chornobyl accident (UNSCEAR 2011). In response to the scientific interest in studying the molecular biology and genomic profile of radiation-related thyroid cancer, the Chornobyl Tissue Bank (CTB) was established including a variety of biological samples from patients with thyroid cancer (Thomas et al. 2000). To date, the CTB has collected biospecimens from about 3,500 individuals who were exposed to ^131^I from the Chornobyl fallout, and from more than 800 sporadic thyroid cancer cases diagnosed in persons who were born after 31 March 1987 and not exposed to ^131^I.

Initially, doses to individuals with samples in the CTB were calculated using the so-called ‘Thyroid Dosimetry 2010 system’ (TD10) methodology (Likhtarov et al. 2013a). This methodology was recently substantially revised and became the ‘Thyroid Dosimetry 2020 system for Ukraine’ (TDU20) methodology. The revision included (i) revision of ^131^I thyroid activities measured in 146,425 individuals, including members of the Ukrainian-American cohort and mothers in the Ukrainian cohort of persons exposed in utero, and the thyroid dosimetry system for the entire Ukraine that was used to calculate the thyroid doses (Masiuk et al. 2021a, b); (ii) estimates of age- and sex-specific thyroid-mass values for residents of the study area (Likhtarov et al. 2013b); and (iii) re-estimation of ^131^I ground deposition densities in Ukraine using a high-resolution meteorological model (Talerko et al. 2020). It is noted that dose estimates for CTB participants, except for those who are members of the Ukrainian-American cohort and the Ukrainian cohort of persons exposed in utero, are based only on limited information on their places of residence at the time of the accident (ATA).

The National Cancer Institute (USA) in collaboration with the V.P. Komisarenko Institute of Endocrinology and Metabolism (IEM, Kyiv, Ukraine) has been conducting a series of studies on the molecular and genomic characterization of thyroid cancer (Morton et al. 2021, 2024). A recently launched study of the effects of age at exposure and radiation dose on molecular changes in thyroid tumors includes 197 individuals from the CTB who were exposed to Chornobyl fallout. Doses for study participants who are members of the Ukrainian-American cohort (*n* = 70) and the Ukrainian cohort of persons exposed in utero (*n* = 3) were previously improved using TDU20 (Masiuk et al. 2022, 2023). However, for the remaining participants (*n* = 124), limited data, i.e., only the place of residence ATA, were available for dose reconstruction. Consequently, to support ongoing molecular studies with reliable dosimetry data, the impact of incorporating information on residential and diet history collected through personal dosimetry interviews on thyroid dose estimates was investigated in the present study.

## Materials and methods

### Study population

Regarding thyroid exposure to ^131^I after the Chornobyl accident, CTB participants were classified into the following groups based on the type of dosimetry-related information available for them (Likhtarov et al. 2013a):

- Group 1 includes members of the Ukrainian-American cohort of individuals exposed during childhood and adolescence whose ^131^I thyroid activity was measured in May–June 1986 and for whom a personal dosimetry interview was conducted to collect individual residence and diet history data.
- Group 2 consists of individuals whose ^131^I thyroid activity was measured in May–June 1986, but with whom no personal dosimetry interview was conducted.
- Group 3 includes individuals who had neither measured ^131^I thyroid activity nor personal dosimetry interviews data. This group is divided into three subgroups, see subsection “CTB dosimetry group 3” below for detail).
- Group 4 consists of individuals who were born between 26 April 1986 and 31 March 1987 and, therefore, were exposed to ^131^I *in utero.* Some of them are members of the Ukrainian cohort of persons exposed *in utero*.

Table 1 provides the distribution of 197 study participants by CTB dosimetry groups, including a subset of 124 individuals from CTB dosimetry groups 2, 3, and 4 for whom personal dosimetry interviews data were not available.

**Table 1:** Distribution of 197 study participants by Chornobyl Tissue Bank (CTB) dosimetry group

CTB dosimetry group	Description	Number of study participants	
		Total	Without dosimetry interview data
1	Ukrainian–American cohort of individuals exposed at age 0–18 y with measured ^131^I thyroid activity and personal dosimetry interview data	70	0
2	Individuals with measured ^131^I thyroid activity but no personal dosimetry interview data	2	2
3	Individuals without measured ^131^I thyroid activity and without personal dosimetry interview data	116	116
4	Individuals who were exposed to ^131^I *in utero*^a^	9	6
Entire study		197	124

^a^ Including three individuals from the Ukrainian cohort of individuals exposed *in utero* for whom personal dosimetry interview data were available

### Individual residence and diet history

The present study includes a subset of 124 out of 197 individuals (62.9% of the total) from CTB dosimetry groups 2 (*n* = 2), 3 (*n* = 116), and 4 (*n* = 6) for whom residence and diet history data were not previously collected (Table 1). Because in the CTB there is only limited information available on details useful for the purpose of dose assessment, i.e., the place of residence ATA and/or at the time of thyroid surgery, there was concern that simply recalculating thyroid doses using TDU20 without using dosimetry questionnaire data would not result in an improvement in dose estimates for these individuals. Therefore, a special study was designed and conducted to collect data on the residence and diet history for these 124 individuals.

**Fig. 1:**
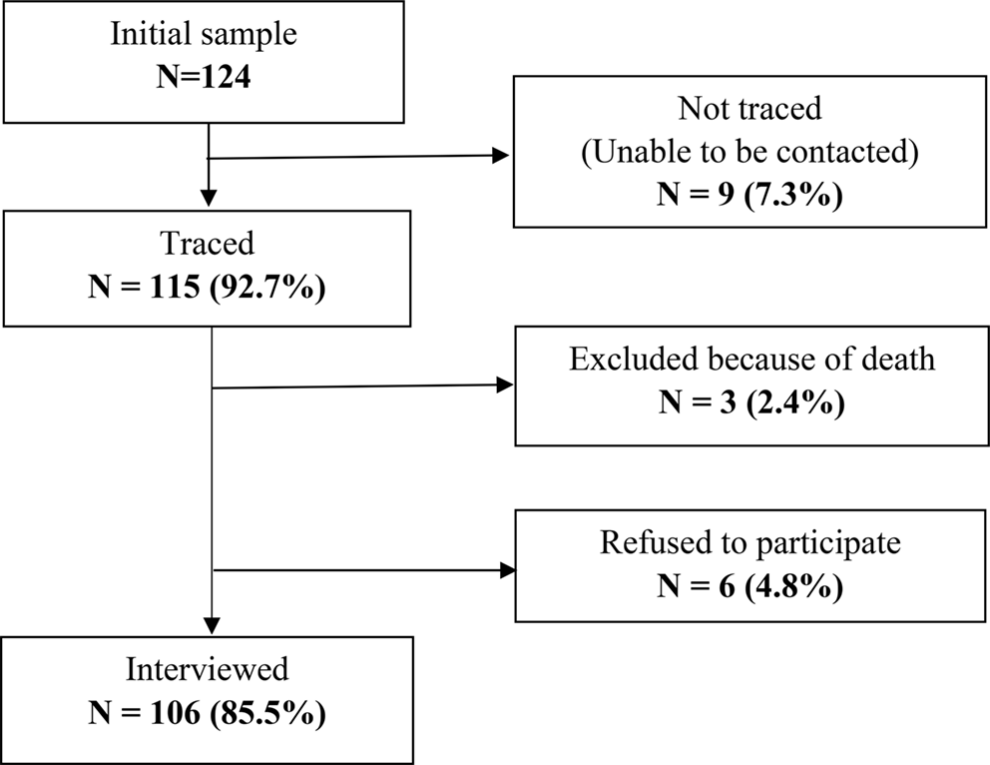
Flow chart of the study participants’ enrollment

Figure 1 shows a flow chart of study participants’ enrollment. The last known address and contact information for these individuals were searched using various sources of information, including the Laboratory of Morphology of the Endocrine system at IEM, the IEM clinical database, blood forms containing information about the address at the time of the Chornobyl accident and a possible change of surname of the CTB participant, medical facilities, and social networks. As a result, 115 individuals (92.7%) could be traced, located, and contacted for conducting a personal dosimetry interview. An invitation letter was sent to everyone explaining the purpose of the personal dosimetry interview and the amount of time and burden expected if they agreed to participate in the study and providing reassurance regarding their privacy and confidentiality concerns as well as a modest payment to compensate their time. The potential study subject was asked to call to schedule an interview. Those who did not respond were followed with a telephone call to confirm their participation or to find out the reason for the refusal. In total, 106 (85.5%) individuals agreed to the interview (Fig. 1). Nine individuals could not be interviewed because of death (*n* = 3, 2.4%) or refusal to participate in the interview (*n* = 6, 4.8%).

Personal dosimetry interviews were conducted during 3 February – 8 April 2023 with 106 individuals from CTB dosimetry groups 2 (*n* = 2), 3 (*n* = 99), and 4 (*n* = 5) by phone or in person (2 individuals resided in Kyiv city). Interviewers were dosimetrists who were specially trained and have gained interview’s experience conducting the interviews in the Ukrainian-American cohort and Ukrainian cohort of persons exposed *in utero*. The questionnaires used in the present study were those previously used in the Ukrainian-American cohort study (Likhtarev et al. 2014) or in the study of the Ukrainian cohort of persons exposed *in utero* (Likhtarov et al. 2011).

The questionnaires included questions about name, date of birth, address on the date of the interview, and questions related to the period 26 April and 14 July 1986 on (i) detailed residence history of the study subject and/or his/her mother for a person exposed in utero and/or an individual who was breastfed; (ii) origin and consumption rates of milk, milk products and leafy vegetables for the study subject (or his/her mother) at each reported place of residence; and (iii) intake of stable iodine by the study subject (or his/her mother) to block the uptake of ^131^I by the thyroid gland.

### Estimation of thyroid doses

The individual doses from ^131^I intake for the CTB subjects from Ukraine were calculated previously by Likhtarov et al. (2013a) using the ‘Thyroid Dosimetry 2010 system’ (TD10), referred in the following as ‘CTBD10’ doses. Revision of CTBD10 thyroid doses using the revised ‘Thyroid Dosimetry 2020 system for Ukraine’ resulted in CTBD23 doses. Approaches to calculate thyroid doses for each CTB dosimetry group are described in the sections below.

#### CTB dosimetry group 1

As mentioned above, the CTB dosimetry group 1 includes members of the Ukrainian–American cohort of individuals exposed in childhood and adolescence, and who developed thyroid cancer. All these individuals were measured for ^131^I thyroid activity in May – June 1986. Individual data on residence history, consumption of milk, milk products and leafy vegetables as well as administration of stable iodine were collected for all cohort members through personal interviews conducted in 2001–2006. Their individual thyroid doses were then estimated based on ^131^I activities in the thyroids and on their responses to questionnaires.

For each study subject *k*, two doses were calculated: (i) a ‘*model-based*’ thyroid dose, $\:{D}_{k}^{model}$, based on the ^131^I thyroid activity, $\:{Q}_{k}^{ecol}\left(t\right)$, that was calculated for any time *t* after the accident using ecological and biokinetic models; and (ii) a ‘*measurement-based*’ thyroid dose, $\:{D}_{k}^{meas}$, based on the ^131^I thyroid activity measured at time $\:{t}_{m}$ after the accident, $\:{Q}_{k}^{meas,\:{t}_{m}}$. The *measurement-based* thyroid dose, which has been used in epidemiological analysis, is more reliable than the *model-based* thyroid dose, because it is based on an individual measurement of ^131^I thyroid activity. The *model-based* thyroid dose ($\:{D}_{k}^{model}$, mGy) for individual *k* was calculated as (Eq. 1):

1 $$\:{D}_{k}^{model}=\frac{{k}_{u}\cdot\:{e}_{th,\:k}^{I-131}}{{M}_{k}}\cdot\:{\int\:}_{0}^{T}{Q}_{k}^{model}\left(t\right)dt$$

where $\:{k}_{u}\:$ = 13.84 is a unit conversion factor (Bq kBq^− 1^ g kg^− 1^ J MeV^− 1^ s d^− 1^ mGy Gy^− 1^); $\:{M}_{k}\:$ is the thyroid mass that corresponds to sex and age of individual *k* (g) (Likhtarov et al. 2013b); $\:{e}_{th,k}^{I-131}$ is the mean energy absorbed in the thyroid gland per decay of ^131^I in the thyroid that corresponds to age of individual *k* (Mowlavi et al. 2011) (MeV); $\:{Q}_{k}^{model}\left(t\right)$ is the variation of the *model-based* activity of ^131^I in the thyroid of individual *k* with time *t* (kBq); $\:T$ = 80 d is the upper limit of integration from ATA on 26 April 1986 (*t* = 0) until 14 July 1986, when ^131^I decayed to a negligible level of 0.1% of the initial deposition.

To calculate the *measurement-based* dose, the *model-based* ^131^I thyroid activity at time of measurement $\:{t}_{m}$, $\:{Q}_{k}^{model}\left(t_{m}\right)$, was replaced in Eq. (1) with the measured activity, $\:{Q}_{k}^{meas,{t}_{m}}$, using the so-called ‘scaling factor’ $\:{SF}_{k}={Q}_{k}^{meas,{t}_{m}}/\:{Q}_{k}^{model}\left(t_{m}\right)$. The scaling factor incorporates the thyroid dose calculation steps, including ecological and biokinetic modeling and questionnaire data, with a $\:{SF}_{k}$- value of 1.0 reflects a perfect agreement between the *model-based* and *measurement-based* doses.

It was assumed that the relative shape of the variation of $\:{Q}_{k}^{model}\left(t\right)$ with time is correct, so that the adjustment at time $\:{t}_{m}$ also applies to any other time after the accident. Under those conditions, the *measurement-based* thyroid dose ($\:{D}_{k}^{meas}$, Gy) was calculated as (Eq. 2):

2 $$\:{D}_{k}^{meas}=\frac{{Q}_{k}^{meas,{t}_{m}}}{\:{Q}_{k}^{model}\left(t_{m}\right)}\cdot\:{D}_{k}^{model}={SF}_{k}\cdot\:{D}_{k}^{model}$$

A detailed description of the dose reconstruction model and its parameters used to calculate thyroid doses for members of the Ukrainian–American cohort can be found elsewhere (Masiuk et al. 2023).

#### CTB dosimetry group 2

The approach to calculate thyroid doses for this group was essentially the same as described above. However, the personal dosimetry interviews data were absent for this group. Therefore, the following assumptions were made to calculate the thyroid doses: (i) place of residence (ATA or at the time of thyroid surgery) recorded at the CTB was assumed as the place of permanent residence during 26 April – 14 July 1986; (ii) to calculate the ^131^I daily intake and *model-based* activity of ^131^I in the thyroid gland of the study individual *k* at time *t*, $\:{Q}_{k}^{model}\left(t\right)$, (Eq. 1), reference age- and gender-dependent consumption rates of milk and leafy vegetables were used (Masiuk et al. 2023); and (iii) there was no administration of stable iodine to block thyroid uptake of ^131^I.

#### CTB dosimetry group 3

The thyroid doses for individuals from this group were calculated using TDU20, which allows to calculate thyroid doses depending on the residence of an individual in a settlement from one of the three levels of information (Masiuk et al. 2021b):

- The first level included 835 settlements of Vinnytsia, Zhytomyr, Kyiv, and Chernihiv Oblasts^1^ where measurements of ^131^I thyroid activity were performed in May and June 1986 among at least 10 residents in each settlement (CTB dosimetry subgroup 3.1).
- The second level included 690 settlements where thyroid measurements were not performed or the number of measured individuals was less than 10, but such measurements were performed in at least five other settlements in a given raion, which is an administrative sub-region of an oblast (CTB dosimetry subgroup 3.2).
- The third level included 28,828 settlements located in those raions where thyroid measurements had not been performed, or where the number of measurements was insufficient. This level includes settlements that were not included in the first and second level (CTB dosimetry subgroup 3.3).

In brief, the model-based thyroid dose, $\:{D}_{k}^{model}$, for individual *k* was calculated using Eq. (1). For individuals for whom personal dosimetry interviews data were not available, the same assumptions as for CTB dosimetry group 2 were used for place of residence, reference diet and stable iodine administration. To calculate the dose for an individual included in the age and gender groups “*a*,* s*” living in the *j*-th settlement, the model-based dose was adjusted using an age- sex- and settlement-specific scaling factor, which calculated as (Eq. 3):

3 $$\:{K}_{a,s,j}^{scal}={D}_{a,s,j}^{\left(l\right)}/{D}_{a,s,j}^{model}$$

where $\:{D}_{a,s,j}^{\left(l\right)}$ is the average thyroid dose for age and gender group “*a*,* s”* for the *j*-th settlement of the *l*-th level (first, second or third) of information described above and in (Masiuk et al. 2021a, b); and $\:{D}_{a,s,j}^{model}$ is the model-based thyroid dose for the same age and gender group “*a*,* s”* and for the same *j*-th settlement.

For individuals for whom personal dosimetry interviews data were not available, thyroid doses for CTB dosimetry group 3 were assumed to be the same as those calculated by Masiuk et al. (2021b). However, for individuals who underwent personal dosimetry interviews, the doses differed from those calculated by Masiuk et al. (2021b), since individual information on places of residence, diet and stable iodine administration was available.

#### CTB dosimetry group 4

For individuals exposed *in utero*, two periods of exposure of the thyroid gland from ^131^I were considered: (i) prenatal exposure of the fetus thyroid gland from intake of ^131^I by the mother during 26 April – 14 July 1986, and (ii) postnatal exposure of the child born before 14 July 1986. Estimation of the prenatal fetus thyroid dose from ^131^I intake by the mother was based on the mother’s thyroid dose using the model from ICRP Publication 88 (ICRP 2001). For individual $\:k$, the *model-based* prenatal dose, $\:{D}_{k,\:fetus}^{model}$ (mGy), was calculated as the sum of the daily doses over all days $\:{t}_{i}\:$ when ^131^I intake by the mother occurred. The maximum value for $\:{t}_{i}$, denoted as $\:{t}_{max}$, was taken to be 80 d (number of days between 26 April and 14 July 1986) or number of days between 26 April 1986 and date of birth for children born before 14 July 1986. The fetal *model-based* thyroid dose was calculated as (Eq. 4):

4 $$\:{D}_{k,fetus}^{model}=\sum\:_{{t}_{i}=0}^{{t}_{max}}\left[{h}_{T}({\tau\:}_{0}+{t}_{i})\cdot\:\underset{{t}_{i}}{\overset{{t}_{i+1}}{\int\:}}{q}_{k,mother}\left(t\right)dt\right]\:,$$

where $\:{h}_{T}\left(\tau\:\right)$ is the fetal thyroid dose coefficient per unit intake of ^131^I by the mother depending on gestational age $\:\tau\:$ (mGy kBq^− 1^) (ICRP 2001); $\:{\tau\:}_{0}$ is the gestational age ATA on 26 April 1986 (d); $\:{q}_{k,mother}\left(t\right)\:$ describes the variation with time of the ^131^I intake by the mother of individual $\:k$ calculated using an ecological model (Likhtarev et al. 2014) (kBq).

To calculate the prenatal thyroid dose of individual $\:k$, $\:{D}_{k,fetus}^{ind}$ (mGy), the *model-based* dose was adjusted by the so-called ‘scaling factor’ for the mother (Eq. 5):

5 $$\:{D}_{k,fetus}^{ind}={D}_{k.fetus}^{model}\cdot\:{K}_{mother}^{scal}$$

where $\:{K}_{mother}^{scal}$ is the scaling factor for the mother to adjust the *model-based* dose defined depending on the availability of ^131^I thyroid activity measured in the mother or, if measurement was not done, depending on residence in settlement belonging to one of the TDU20 levels as described above in the Section ‘CTB dosimetry group 3 of the cohort member’ (unitless).

For individuals born before 14 July 1986, the postnatal thyroid dose from their ^131^I intake was also calculated in the same way as for CTB dosimetry group 3. For breastfed individuals, the ^131^I activity concentration in breast milk was estimated using the ecological model and data on the mother’s residences and food consumptions during breastfeeding. The variation with time of the ^131^I intake by the child was estimated using the ecological model and data on diet for the child.

The present study included three individuals from the Ukrainian cohort of those exposed *in utero* for whom personal dosimetry interview data were available. A detailed description of the dosimetry models used to calculate thyroid doses for individuals exposed *in utero* can be found elsewhere (Masiuk et al. 2022).

### Assessment of uncertainties in doses

To assess dose uncertainties, a Monte Carlo method was used to calculate 1,000 individual stochastic doses from ^131^I intake for each studied individual. The classical and Berkson types of errors were evaluated separately because their influences on estimates of radiation-related risk of thyroid cancer following exposure to ^131^I were considerably different (Masiuk et al. 2016, 2017). Dose realizations for the same cohort member $\:k$, from $\:{D}_{1,k}$ to $\:{D}_{1000,k}$, represent 1,000 *individual stochastic* thyroid doses of this individual. The arithmetic means of 1,000 *individual stochastic* thyroid doses is called hereinafter as ‘thyroid dose’ of this individual. A detailed description of model parameters with separation of the sources of classical and Berkson-type errors for dose reconstruction models used to calculate doses for individuals belonging to different CTB dosimetry groups can be found elsewhere (Drozdovitch et al. 2023; Likhtarev et al. 2014; Masiuk et al. 2021b, 2022, 2023).

## Results and discussion

### Thyroid dose estimates

The arithmetic mean of thyroid doses from ^131^I intake estimated in the present study was 510 mGy, while the median was 81 mGy. A rather wide range of thyroid doses was observed among study participants: doses varied from zero to 11.9 Gy. Doses more than 0.5 Gy were estimated for 40 individuals (20.3% of the total) while 21 (10.7%) individuals received doses more than 1.0 Gy. More than half of study participants, 109 out of 197, received doses less than 100 mGy.

Table 2 shows the thyroid doses from ^131^I intake to study participants according to the CTB dosimetry group, the oblast of residence ATA, and the type of the settlement of residence ATA. As expected, the highest doses were realized among individuals of the CTB dosimetry groups 1 (mean dose = 1,160 mGy) and 2 (2,685 mGy), since all these individuals were measured for ^131^I thyroid activity, and these measurements were performed in most contaminated areas. The highest doses (mean = 950 mGy and median = 265 mGy) were received in Zhytomyr Oblast, the most contaminated region in Ukraine. Thyroid doses received in rural settlements were higher than those in urban settlements, 715 mGy vs. 285 mGy for the arithmetic mean and 225 mGy vs. 39 mGy for the median, respectively (Table 2). This can be explained by the higher contamination of cow’s milk (a major source of ^131^I intake) with ^131^I in rural settlements compared to urban areas. This was also observed in other populations exposed to Chornobyl fallout (Drozdovitch et al. 2013; Masiuk et al. 2023).

**Table 2:** Thyroid doses from ^131^I intakes by selected groups for 197 study participants from Chornobyl Tissue Bank (CTB) dosimetry groups 1, 2, 3, and 4; N – number of individuals

Parameter	N	^131^I thyroid dose (mGy)		
		Mean	Median	Range
CTB dosimetry group				
– Group 1	70	1,160	350	14–11,910
– Group 2	2	2,685	2,685	1,740–3,630
– Group 3	116	115	40	0–1,460
– Group 4	9	96	35	0–310
Place of residence ATA				
– Zhytomyr Oblast	38	950	265	15–8,640
– Kyiv Oblast	94	305	38	2.9–11,910
– Chernihiv Oblast	59	610	255	8.3–5,380
– Others	6	21	27	0–39
Type of settlement of residence				
– Rural	104	715	225	0–8,640
– Urban	93	285	39	2.9–11,910
Entire study population	197	510	81	0–11,910

Table 3 provides characteristics of 15 study participants with thyroid doses from ^131^I intake of 2 Gy and more. They resided ATA in settlements with ^131^I ground deposition density ranging from 0.14 MBq m^− 2^ to 254 MBq m^− 2^. Three individuals were evacuated from the 30-km zone around the Chornobyl NPP on 27–28 April 1986, and seven individuals voluntary relocated from highly contaminated settlements between 3 May and 20 June 1986. All of them consumed fresh cow’s milk, except two individuals: one consumed goat milk and another was breastfed. All study participants with thyroid doses from ^131^I intake of 2 Gy and more were measured for ^131^I thyroid activity. Table 3 also provides the scaling factor, $\:{SF}_{k}$, values that is an indicator of the credibility of dose estimates (Eq. 2). Previous analysis of thyroid doses of the Belarusian-American cohort showed that a deviation of $\:{SF}_{k}$-values from 1.0 (perfect agreement between the *model-based* and *measurement-based* doses) by more than 100 times reflected a potentially suspicious thyroid dose (Kukhta et al. 2021). The $\:{SF}_{k}$-values varied between 0.42 and 12, suggesting that thyroid doses from ^131^I intake for most exposed individuals included in this study were estimated with a high degree of accuracy and reliability.

**Table 3:** Characteristics of 15 study participants with thyroid doses from ^131^I intake more than 2 Gy. CTB - Chornobyl Tissue Bank; ATA – at the time of the accident

Individual	CTB dosimetry group	Age ATA (y)	Raion or settlement of residence ATA	^131^I ground deposition density^a^ (MBq m^− 2^)	Milk consumption^b^ (L d^− 1^)	Date of first relocation in 1986	Date of ^131^I thyroid activity measurement in 1986	^131^I activity measured in thyroid (kBq)	Thyroid dose from ^131^I intake (Gy)	Scaling factor, $\:{SF}_{k}$
A	1	3	Pripyat town^c^	88	0.35^d^	27 April	24 May	133	11.9	3.1
B	1	1	Narodychi	9.7	1.0	25 May	30 May	56	8.6	1.6
C	1	0	Chernihiv	0.14	0.5^e^	‒ ^f^	26 May	38	5.4	2.0
D	1	1	Pripyat town^c^	88	0.5^d^	27 April	10 June	12	4.9	3.1
E	2	2	Narodychi	6.3	0.25	26 May	27 May	44	3.7	3.4
F	1	2	Bragin^c^ (Belarus)	254	0.3	28 April	27 May	18	3.5	0.42
G	1	15	Poliske	21	1.0	12 May	22 May	330	3.5	7.2
H	1	4	Chernihiv	1.6	0.25	‒	19 May	141	3.4	2.0
I	1	7	Narodychi	5.5	1.0	19 May	17 May	220	2.8	1.4
J	1	2	Ripky	0.29	0.5	‒	17 May	79	2.7	5.4
K	1	0	Chernihiv	0.15	0.8^g^	‒	27 May	17	2.4	2.4
L	1	1	Narodychi	2.2	1.0	‒	17 May	50	2.3	0.98
M	1	9	Chernihiv	0.43	0.4	3 May	20 May	129	2.2	12
N	1	6	Poliske	9.4	1.0	20 June	8 June	14	2.2	1.1
O	1	10	Chernihiv	1.4	1.0	16 May	28 May	90	2.0	1.1

^a^ At the place of residence ATA (Talerko et al. 2020)

^b^ Consumption of private cow’s milk unless otherwise indicated

^c^ Settlement was evacuated from the 30-km zone around the Chornobyl NPP

^d^ Milk consumption is given for the settlement of relocation since there was no intake of ^131^I with milk in the town of Pripyat

^e^ Goat milk

^f^ There was no relocation from the place of residence ATA between 26 April and 14 July 1986

^g^ Breast milk

### Comparison of revised thyroid doses with previous estimates

**Fig. 2:**
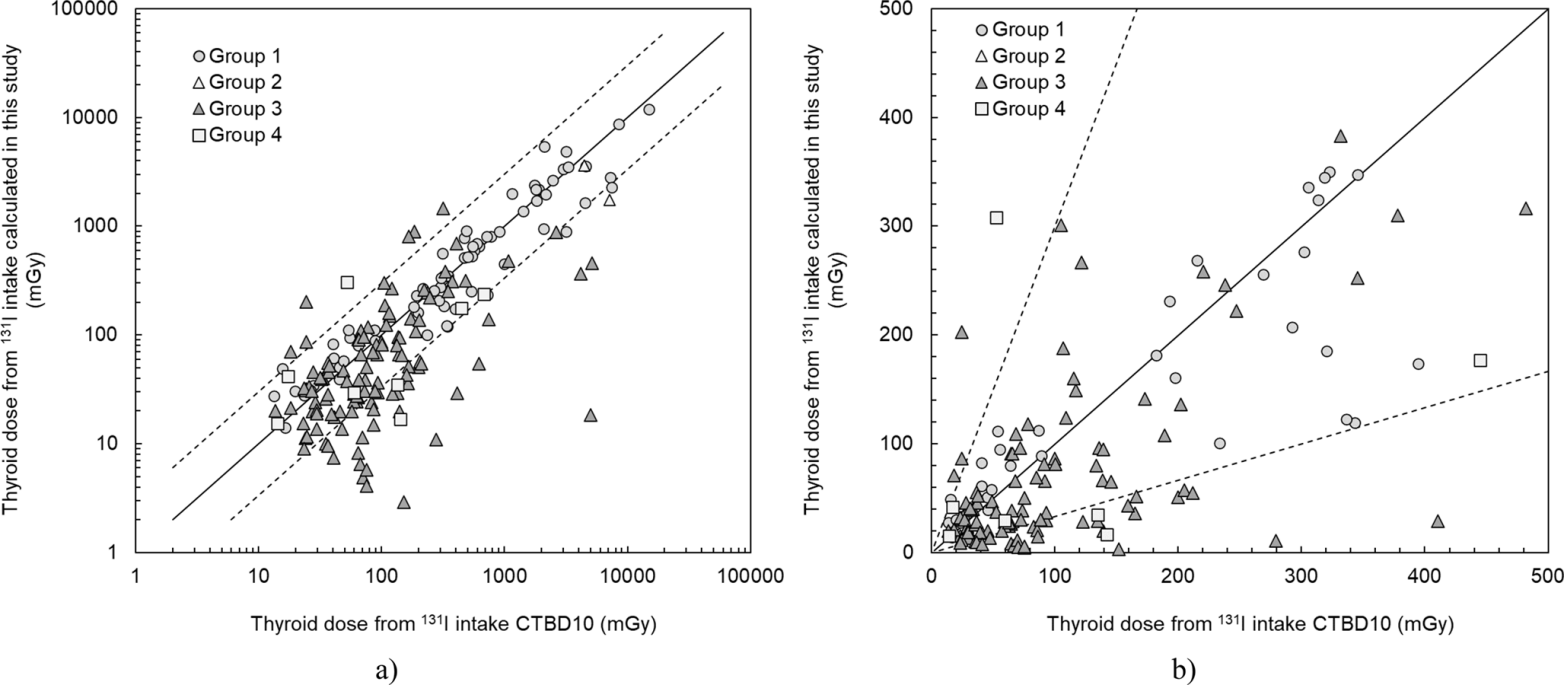
Comparison of the arithmetic means of 1,000 *individual stochastic* thyroid doses from ^131^I intake, CTBD23 vs. CTBD10. Solid line indicates agreement between doses, while dashed lines indicate a factor of 3 difference. (**a**) logarithmic scale for the entire dose range, (**b**) linear scale for doses less than 500 mGy

Figure 2 compares the doses from ^131^I intake calculated in this study (CTBD23) with those estimated previously (CTBD10) by Likhtarov et al. (2013a, b). The difference between the two doses was mainly caused by the following reasons: First, the revision of ^131^I thyroid activity measured in 146,425 individuals, including members of the Ukrainian-American cohort (CTB dosimetry group 1) and mothers of the members of the Ukrainian cohort of persons exposed in utero (CTB dosimetry group 4) done by Masiuk et al. (2021a) during the development of TDU20. This revision was necessary to correct improper measurement geometry and evaluate true values of calibration coefficients for unchecked thyroid detectors used to measure ^131^I activity in some individuals. Second, the use of TDU20 and individual residence and diet history data collected in this study for individuals from CTB dosimetry groups 2, 3, and 4 (Masiuk et al. 2021b).

Table 4 compares the mean and median values of the two doses, CTBD23 and CTBD10, by CTB dosimetry groups. The arithmetic mean of CTBD23 doses for the entire study was about 25% less than that estimated in CTBD10, 510 mGy vs. 700 mGy, respectively, while the global median of CTBD23 doses was reduced by more than 30% compared to CTBD10, 81 mGy vs.120 mGy, respectively. The largest reduction in CTBD23 doses, by a factor of about 2, was observed for individuals from CTB dosimetry groups 2, 3, and 4, who previously did not have individual questionnaire data. A detailed comparison of doses for these individuals is given in the section below.

**Table 4:** Comparison of thyroid doses from ^131^I intake by Chornobyl Tissue Bank (CTB) dosimetry group, CTBD23 vs. CTBD10. N – number of individuals

CTB dosimetry group	N	Thyroid dose from ^131^I intake (mGy)					
		Mean			Median		
		CTBD23	CTBD10	CTBD23/ CTBD10	CTBD23	CTBD10	CTBD23/ CTBD10
1	70	1,160	1,345	0.86	350	430	0.81
2	2	2,685	5,770	0.47	2,685	5,770	0.47
3	116	115	265	0.43	40	75	0.53
4	9	96	170	0.56	35	60	0.58
Entire study	197	510	700	0.73	81	120	0.68

### Individual behavior and dietary data

Table 5 shows the descriptive statistics for individual behaviour and dietary data used to calculate the following thyroid doses for 106 individuals who were interviewed in the present study:

**Table 5:** Descriptive statistics for individual behavior and dietary data used to calculate CTBD23_No_ and CTBD23_Qx_ doses. CTB - Chornobyl Tissue Bank

Behavior and dietary data	Used in calculation of doses	
	CTBD23_No_	CTBD23_Qx_
Residential history		
– Percentage of study participants who reported 1 relocation	0%	46%
– Percentage of study participants who reported 2 relocations	0%	15%
Percentage of breastfed study participants	13%	6.7%
Consumption of milk from privately owned cows		
– Percentage of consumers	87%	39%
– Daily consumption ^a^ (L/d)	0.21 ± 0.16 (0.052–0.65)	0.30 ± 0.30 (0–1.3)
– Percentage of consumers who change milk consumption’s habits	0%	73%
Consumption of milk from commercial trade network		
– Percentage of consumers	87%	49%
– Daily consumption (L/d)	0.13 ± 0.06 (0.032–0.22)	0.20 ± 0.18 (0–1.0)
– Percentage of consumers who change milk consumption’s habits	0%	73%
Consumption of milk from privately owned goat		
– Percentage of consumers	0%	4.1%
– Daily consumption (L/d)	0	0.30 ± 0.05 (0.25–0.35)
– Percentage of consumers who change milk consumption’s habits	0%	25%
Consumption of dairy products		
– Percentage of consumers	87%	63%
– Daily consumption (g/d)	33 ± 7 (19–44)	68 ± 37 (0–200)
– Percentage of consumers who change consumption’s habits	0%	44%
Consumption of leafy vegetables		
– Percentage of consumers	87%	63%
– Daily consumption (g/d)	18 ± 8 (7–35)	18 ± 16 (1–57)
– Percentage of consumers who change consumption’s habits	0%	1.6%
Percentage of study participants with administration of stable iodine	0%	6.6%

^a^ Arithmetic mean ± standard deviation, the range of consumption rates is given in parentheses

- CTBD23_No_ doses that were calculated using TDU20 without individual questionnaire data. The following behaviour and dietary data were used: permanent residence in the settlement of residence ATA and reference age- and gender-dependent consumption rates of milk, dairy products and leafy vegetables; stable iodine administration was not considered; and
- CTBD23_Qx_ doses that were calculated using TDU20 and individual questionnaire data on residence and diet history and stable iodine administration.

Regarding residential history, 49 out of 106 individuals (46% of the total) reported relocation from the place of residence ATA, while 16 out of 106 individuals (15% of the total) reported two relocations. According to the questionnaire data, the fraction of consumers of milk was lower than that established in TDU20, 39% vs. 87% and 49% vs. 87%, for milk from privately owned cows and from commercial trade network, respectively. However, the arithmetic mean of consumption rates of milk reported during personal interviews was higher than that established in TDU20, 0.30 L d^− 1^ vs. 0.21 L d^− 1^ and 0.20 L d^− 1^ vs. 0.13 L d^− 1^, for milk from privately owned cows and from commercial trade network, respectively. TDU20 did not consider milk consumption from privately owned goats since the fraction of consumers was very low. Indeed, only 4.1% of the study participants reported consuming goat’s milk. According to TDU20, breastfeeding was assumed for all 14 individuals aged less than 1 y ATA; however, this was reported during personal interviews only for seven of them. The fraction of individuals reporting consumption of dairy products and leafy vegetables was slightly lower than that assigned in TDU20, 63% vs. 87%, for both. TDU20 did not account for the administration of stable iodine to block the uptake of radioactive iodine by the thyroid gland. However, seven out of 106 individuals (6.6% of the total) reported having taken stable iodine during the first month after the accident.

### Impact of individual questionnaire data on doses

To assess the impact of using individual questionnaire data, the two sets of doses, CTBD23_No_ and CTBD23_Qx_, were compared for 106 individuals who were interviewed in this study. CTBD23_Qx_ doses were found to be lower than CTBD23_No_ doses, 170 mGy vs. 245 mGy for the arithmetic mean and 39 mGy vs. 105 mGy for the median, respectively.

To evaluate the main reasons for changes in thyroid doses, the following dose ratios were analyzed: (i) CTBD23_No_ / CTBD10 vs. CTBD10 to assess the impact of using TDU20 and (ii) CTBD23_Qx_ / CTBD23_No_ vs. CTBD23_No_ to assess the impact of using individual questionnaire data collected in this study. Table 6 shows the distribution of study participants with different ranges of these ratios, reflecting increases or decreases in thyroid doses due to application of the revised methodology and use of questionnaire data. Application of TDU20 resulted mainly in 1–3-fold changes in thyroid doses in 99 out of 104 (95.2% of the total) individuals mainly in the low (< 100 mGy, *n* = 54) and medium low (100–300 mGy, *n* = 32) dose ranges. The use of questionnaire data was the main reason for significant (a factor more than 3) changes in doses in the medium low and medium dose ranges (100–1,000 mGy) in 32 from 48 individuals (66.7% of the total), while the use of TDU20 caused the same change in only 1 from 42 individuals (2.4%). In the high (> 1,000 mGy) dose range, changes in doses within a factor 3–10 due to each of the reason considered were observed in 2 from 6 individuals (33.3%) (Table 6).

**Table 6:** Reasons for changes in thyroid doses among individuals who were interviewed in the present study. CTB - Chornobyl Tissue Bank

Thyroid dose range^a^ (mGy)	Number of individuals for whom thyroid doses were changed within					
	factor^b^ of 1–3 due to		factor of 3–10 due to		factor of 10–30 due to	
	Methodology^c^	questionnaire data^d^	methodology	questionnaire data	methodology	questionnaire data
*Increase in doses*						
< 100	45	5	1	1	1	‒
100–300	16	2	‒	7	‒	‒
300–1000	4	‒	‒	1	‒	‒
1000+	‒	‒	‒	‒	‒	‒
Entire Study	65	7	1	9	1	‒
*Decrease in doses*						
< 100	9	31	‒	11	‒	2
100–300	16	14	1	12	‒	2
300–1000	5	‒	‒	6	‒	4
1000+	4	4	2	2	‒	‒
Entire Study	34	49	3	31	‒	8
*Both directions*						
Entire Study	99	56	4	40	1	8

^a^ Thyroid dose range is given for CTBD10 doses for column ‘methodology’ and for CTBD23_No_ doses for column ‘questionnaire data’

^b^ Changing, increasing or decreasing in dose by the specified number of times

^c^ Two study participants from 106 interviewed are not included in the analysis since (i) for one individual all doses, CTBD10, CTBD23_No_ and CTBD23_Qx_, were zero and (ii) for one individual CTBD23_No_ dose changed not because of use of TDU20, but because the CTB dosimetry group was reassigned from 2 to 3 in CTBD23_No_ dose calculation, because the ^131^I thyroid activity measurement was previously assigned to that individual by mistake

^d^ Two study participants from 106 interviewed are not included in the analysis since (i) for one individual all doses, CTBD10, CTBD23_No_ and CTBD23_Qx_, were zero and (ii) for one individual CTBD23_Qx_ dose was zero

**Fig. 3:**
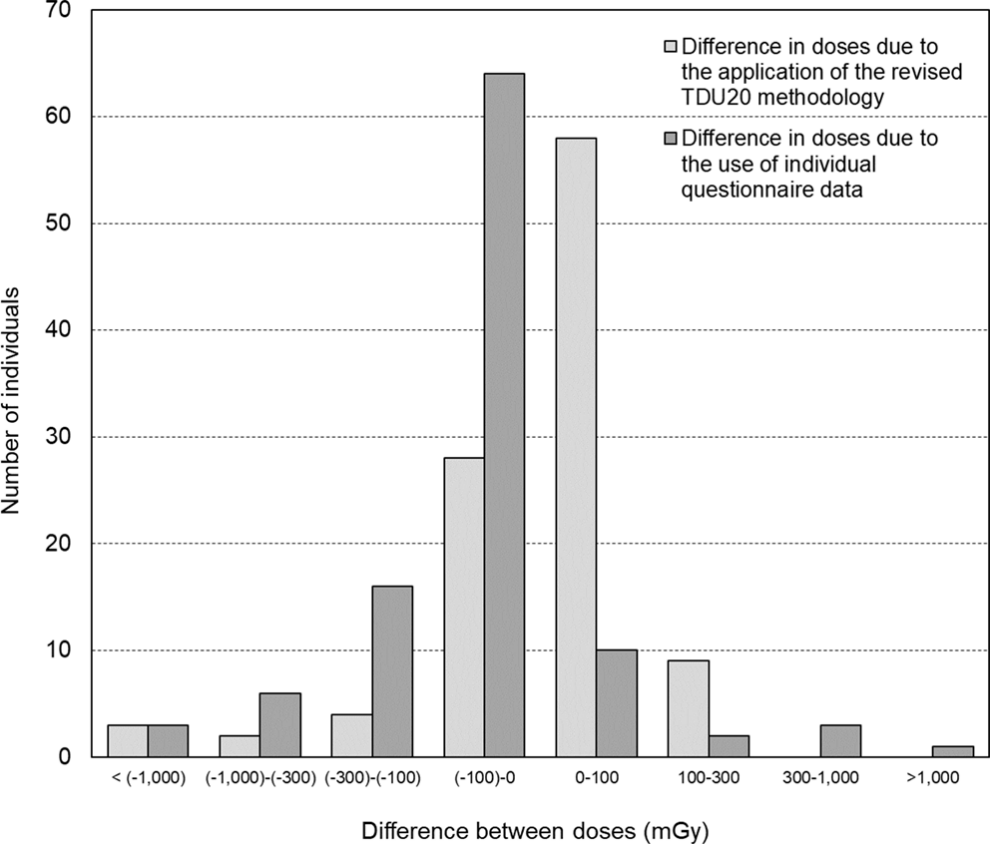
Change in absolute dose values due to the application of the revised methodology (difference between CTBD23_No_ and CTBD10) and the use of individual questionnaire data (difference between CTBD23_Qx_ and CTBD23_No_)

Figure 3 shows a histogram of the change in absolute dose values due to the use of the revised methodology (difference between CTBD23_No_ and CTBD10) and the use of questionnaire data (difference between CTBD23_Qx_ and CTBD23_No_). Dose changes of more than 100 mGy were observed in 18 out of 104 (17.3%) individuals due to the use of TDU20 and in 31 out of 104 (29.8%) individuals due to the use of questionnaire data.

Table 7 provides the reasons for the changes in CTBD23_Qx_ doses compared to CTBD23_No_ doses for a sample of 15 individuals with a notable difference between the two doses. The inclusion of residence history data led primarily to a reduction in individual doses as information collected during personal interviews on evacuation or relocation from a highly contaminated settlement of residence ATA to low or non-contaminated areas became available (subjects C1, D1, F1, G1, H1 and J1). In contrast, ascertaining residence history resulted in a significant increase in thyroid dose for subject K1 from 33 mGy to 205 mGy. The CTBD23_No_ dose of 33 mGy for subject K1 was calculated using CTB information on residence ATA in Kyiv city, while it was found that this individual lived ATA in a high contaminated settlement in Zhytomyr Oblast and moved to the city of Kyiv on 6 May 1986. Accounting for dietary history data resulted either in increase or decrease in individual dose-values depending on the extent to which reported milk origin and consumptions ATA differed from those used in the previous dose reconstruction model (subjects A1, B1, C1, I1, and M1). Subjects O1 reported consumption of goat milk that led to a significant increase in thyroid dose since the ^131^I concentration in goat milk is almost 10 times greater than that in cow’s milk (Fesenko et al. 2007; Voigt et al. 1989). Reported administration of stable iodine ATA resulted in a more than two-fold reduction in thyroid dose from ^131^I intake for subject E1.

**Table 7:** Reasons for the changes in CTBD23_Qx_ doses compared to CTBD23_No_ doses for 15 individuals with a notable difference between the two doses. CTB - Chornobyl Tissue Bank; ATA – at the time of the accident

Subject	Thyroid dose from ^131^I intake (mGy)			Reason for difference^a^	^131^I ground deposition density^b^ (MBq m^− 2^) at residence ATA that was used to calculate		Milk^c^ consumption (L d^− 1^) ATA that was used to calculate	
	CTBD23_No_	CTBD23_Qx_	Difference		CTBD23_No_	CTBD23_Qx_	CTBD23_No_	CTBD23_Qx_
A1	2,550	885	-1,665	Diet	2.4	2.4	0.51	0.13
B1	1,590	460	-1,130	Diet	6.3	6.3	0.68	0.4 (cessation on 5 May)
C1	1,490	370	-1,120	Residence	6.3	6.3 (relocated on 10 May)	0.68	0.13 (cessation on 10 May)
D1	770	54	-716	Residence and diet	88	88 (evacuated on 28 April)	0.32	0.32 (cessation on 28 April)
E1	1,010	475	-535	Other^d^	1.8	1.8	0.34	0.25
F1	330	11	-319	Residence	21	0.45	0.46	0.04
G1	330	19	-311	Residence	1.3	1.3 (relocated on 29 April)	0.68	0.5 (cessation on 29 April)
H1	130	0	-130	Residence	0.45	0	0.8^e^	0.8^e^
I1	115	3	-112	Diet	0.28	0.28	0.51	0
J1	110	4	-106	Residence	0.45	0.45 (relocated on 29 April)	0.33	0.5 (cessation on 29 April)
K1	33	205	172	Residence	0.45	6.3 (relocated on 6 May)	0.18	0.25 (cessation on 6 May)
L1	280	690	410	Diet	0.26	0.26	0.8^e^	0.5
M1	190	810	620	Diet	0.45	0.45	0.34	1.4
N1	140	890	750	Residence and diet	0.45	0.45 (relocated on 29 April)	0.8^e^	0.35^f^
O1	335	1,460	1,125	Diet	1.0	1.0	0.51	0.25^f^

^a^ ‘Diet’ refers to the diet history data and ‘Residence’ refers to the residence history data on 26 April – 14 July 1986 that were collected during the personal dosimetry interviews conducted during this study

^b^ According to Talerko et al. (2020)

^c^ Cow’s milk unless otherwise indicated

^d^ Stable iodine administration on 26 April 1986

^e^ Breast milk

^f^ Goat milk

**Fig. 4:**
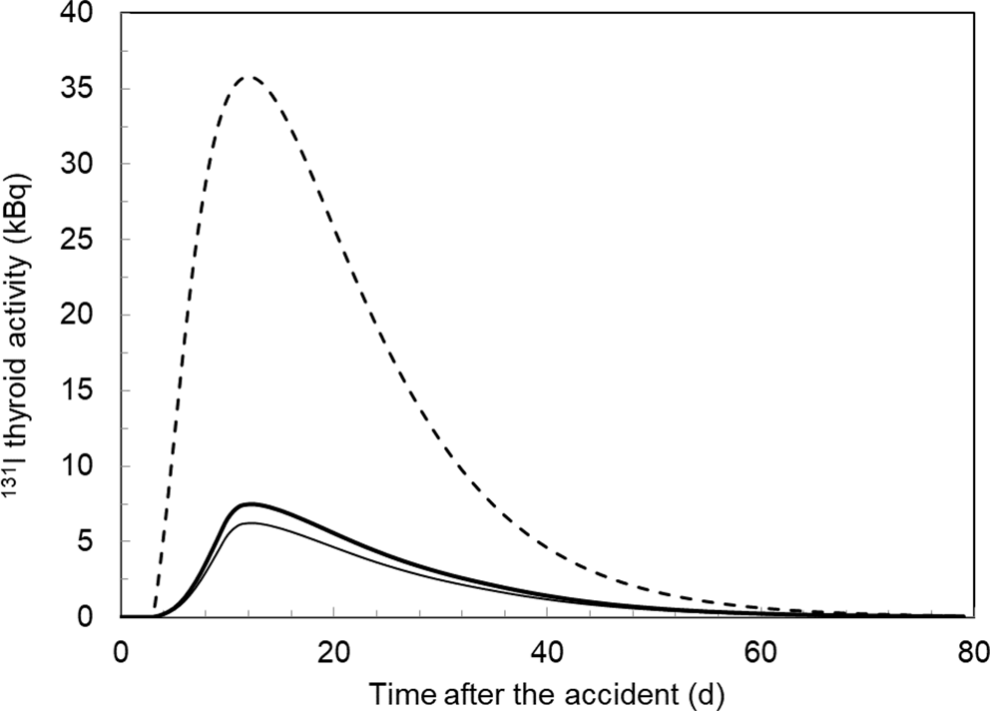
Variation with time of ^131^I thyroid activity calculated for individual N1 (see Table 7) using TD10 (thin line), TDU20 without questionnaire data (thick line), and both TDU20 and individual questionnaire data (dashed line)

Figure 4 shows the variation with time of ^131^I thyroid activity calculated for subject N1 using (i) TD10, (ii) TDU20 without questionnaire data, and (iii) both TDU20 and individual questionnaire data, which corresponds to CTBD10, CTBD23_No_ and CTBD23_Qx_ doses, respectively. Since the area under the curve of ^131^I thyroid activity is proportional to the thyroid dose, the figure demonstrates that there is a significant increase in CTBD23_Qx_ dose, because consumption of goat milk was reported for this individual instead of breastfeeding after relocation on 29 April 1986. Note that TD10 and TDU20 did not consider any changes in consumption habits as well as the relocation, but assumed that this individual was breastfed between 29 April and 14 July 1986 and resided in the same settlement. Therefore, there is practically no difference between CTBD10 and CTBD23_No_ doses. This example clearly shows that the use of individual residence and dietary data was the main reason for the significant, more than 6-fold (from 140 mGy to 890 mGy), increase in N1 thyroid dose.

### Uncertainties in doses

Sets of multiple 1,000 *individual stochastic* doses were calculated for each study participant. The fitted distribution of *individual stochastic* doses for each individual was found to be approximately lognormal and the geometric standard deviation (GSD) of that distribution was used to characterize the overall uncertainty for every individual studied. For CTBD23, the GSD ranged from 1.3 to 5.3 with an overall arithmetic mean of 2.7 and a median of 3.0 across all study participants, while for previous CTBD10 dose estimates, the GSD ranged from 1.3 to 4.5, with a mean of 2.5 and a median of 2.6.

As expected, the highest GSDs were associated with the doses calculated for individuals who were not measured, i.e., CTB dosimetry groups 3 and 4, where the GSD ranged from 2.6 to 5.3 with a mean of 3.4 and a median of 3.3, while for previous CTBD10 dose estimates the GSD ranged from 1.5 to 4.5 with a mean of 3.1 and median of 3.2. The lowest GSDs were associated with the CTBD23 doses calculated for 70 individuals from CTB dosimetry group 1 who are members of the Ukrainian-American cohort and who were measured for ^131^I thyroid activity (mean GSD = 1.6, median GSD = 1.4, GSD range from 1.3 to 3.3). For the CTBD10 doses the corresponding numbers are mean GSD = 1.5, median GSD = 1.5, range from 1.3 to 3.7, respectively. As was shown in (Drozdovitch et al. 2015; Masiuk et al. 2023), the calibration of the *model-based* dose by the *model-based *^131^I thyroid activity has substantially reduced all sources of errors, because the same values of parameters of the *model-based* model were used during calculation of the given dose to estimate the *model-based*^131^I thyroid activity and the *model-based* dose. GSDs for individual doses practically did not change except for five individuals, and these changes are related to the revision of measured ^131^I thyroid activity and introduction of new sources of errors (see Masiuk et al. 2021a for details).

Individual GSD-values of CTBD23 doses increased compared to CTBD10 doses in study participants from the CTB dosimetry group 4: mean GSD increased from 3.0 to 3.7, and median GSD increased from 3.0 to 3.5, respectively. This increase was due to the inclusion of new sources of errors in the calculation of 1,000 individual stochastic doses for individuals exposed *in utero*, such as those for the fetal thyroid dose coefficient and gestation age ATA (Masiuk et al. 2022).

### Strengths and limitations of the present study

The strength of the present study is the use of the revised TDU20 thyroid dosimetry system. Another strength of the study is the use of individual data of residence and diet history to calculate thyroid doses from ^131^I intake for most study participants (179 out of 197, 90.9% of the total).

However, it should be noted that this study has also some limitations:

1. Reliability of information collected in 2023, i.e., 37 years after the accident, during personal dosimetry interviews may be low due to the respondent’s poor memory. However, as was shown in the Belarusian-American cohort of individuals exposed during childhood and adolescence, memory recall of the cohort participants of residence history is quite reliable with the reproducibility of this information of 88–92%, while memory about consumption of milk and milk products was slightly worse with a reproducibility around 55–80% (Drozdovitch et al. 2016).
2. Lack of residence and dietary history data for 18 study participants who were not interviewed in the present study. Consequently, it was not feasible to estimate the thyroid doses for these individuals, and therefore their doses represent age- and sex-averaged *model-based* doses for the settlement of residence. Difference in residential history and diet between those assumed in the study (permanent residence and imputed age- and sex-averaged consumption rates) and the ‘true’ but unknown data for these individuals without personal interviews may lead, as it was shown in the present study, to substantial overestimation or underestimation of *model-based* thyroid doses.

## Conclusions

This paper presents improved estimates of thyroid doses from ^131^I intake based on revised methodology and individual residence and diet history for CTB subjects who are enrolled in an ongoing study on effects of age at exposure and radiation dose on molecular changes in thyroid tumors after the Chornobyl accident. The present study included a rather limited sample of exposed Ukrainians enrolled in CTB, 197 out of about 3,800 (5.2% of the total). The study was able to evaluate the impact of TDU20 as well as the use of questionnaire data collected through personal dosimetry interviews on previously estimated thyroid dose-values. It was clearly shown that the use of individual residence and diet history data was the main reason for significant (a factor more than 3) changes in thyroid doses, especially in the 100–1,000 mGy dose range, for around half of individuals included in CTB dosimetry groups 2, 3, and 4, while the impact of applying TDU20 was less pronounced. CTB dosimetry groups 2, 3, and 4 include more than 90% of around 3,800 exposed individuals currently enrolled in the entire CTB. Consequently, it is concluded that, when conducting special studies aimed at elucidating possible associations between the thyroid dose and important demographic, clinical, pathological, molecular genetic indicators, it is essential to conduct personal dosimetry interviews to collect data on the residence and diet history of study participants. This will allow to provide more realistic individual dose estimates than without including those interviews. Another lesson learned from the present study is that whenever a radiation accident occurs, it is important to ask affected individuals through health and radiation safety authorities to keep records of their own behaviour and diet, and, if possible, those of their children.

## Data Availability

Data will be available at the DCEG Publication Data Repository under Data Sharing Plan #dmsp00409.v3.

## Footnotes

1 An oblast is the largest administrative unit in Ukraine with a typical size of 10,000–30,000 km^2^ and a population of 1.0–1.5 million persons. Each oblast is sub-divided into about 20 raions of about similar size and population.
